# Phosphatidylserine Exposing Extracellular Vesicles in Pre-eclamptic Patients

**DOI:** 10.3389/fmed.2021.761453

**Published:** 2021-11-03

**Authors:** Sanja Lalic-Cosic, Violeta Dopsaj, Mirjana Kovac, Vesna Mandic-Markovic, Zeljko Mikovic, Fariborz Mobarrez, Aleksandra Antovic

**Affiliations:** ^1^Department of Medical Biochemistry, Faculty of Pharmacy, University of Belgrade, Belgrade, Serbia; ^2^Haemostasis Department, Blood Transfusion Institute of Serbia and Faculty of Medicine, University of Belgrade, Belgrade, Serbia; ^3^Gynaecology and Obstetrics Clinic “Narodni Front”, Faculty of Medicine, University of Belgrade, Belgrade, Serbia; ^4^Department of Medical Sciences, Uppsala University, Uppsala, Sweden; ^5^Department of Medicine, Division of Rheumatology, Karolinska Institutet and Rheumatology, Karolinska University Hospital Stockholm, Stockholm, Sweden

**Keywords:** extracellular vesicles, endogenous thrombin potential, overall haemostatic potential, fibrin structure, endothelial dysfunction, pre-eclampsia

## Abstract

**Background:** Pre-eclampsia (P-EC) is associated with systemic inflammation, endothelial dysfunction and hypercoagulability. The role of extracellular vesicles (EVs) in coagulation disturbances affecting the development and severity of P-EC remains elusive. We aimed to evaluate the concentration of EVs expressing phosphatidylserine (PS) and specific markers in relation to the thrombin and fibrin formation as well as fibrin clot properties, in pregnant women with P-EC in comparison to healthy pregnant women of similar gestational age.

**Methods:** Blood samples of 30 pregnant women diagnosed with P-EC were collected on the morning following admission to hospital and after delivery (mean duration 5 days). The concentration of the PS-exposing EVs (PS+ EVs) from platelets (CD42a^+^, endothelial cells (CD62E^+^), and PS+ EVs expressing tissue factor (TF) and vascular cell adhesion molecule 1 (VCAM-1) were measured by flow cytometry. Further phenotyping of EVs also included expression of PlGF. Markers of maternal haemostasis were correlated with EVs concentration in plasma.

**Results:** Preeclamptic pregnancy was associated with significantly higher plasma levels of PS+ CD42a^+^ EVs and PS+ VCAM-1^+^ EVs in comparison with normotensive pregnancy. P-EC patients after delivery had markedly elevated concentration of PS+ CD42a^+^ EVs, CD62E^+^ EVs, TF^+^ EVs, and VCAM-1^+^ EVs compared to those before delivery. Inverse correlation was observed between EVs concentrations (PS+, PS+ TF^+^, and PlGF^+^) and parameters of overall haemostatic potential (OHP) and fibrin formation, while PS+ VCAM-1^+^ EVs directly correlated with FVIII activity in plasma.

**Conclusion:** Increased levels of PS+ EVs subpopulations in P-EC and their association with global haemostatic parameters, as well as with fibrin clot properties may suggest EVs involvement in intravascular fibrin deposition leading to subsequent microcirculation disorders.

## Introduction

Pre-eclampsia (P-EC) is a pregnancy-specific multisystem disorder associated with high perinatal and maternal morbidity and mortality rates, and at the same time linked to long-term health consequences for mothers and their offspring (1–3). The root cause of P-EC is considered to be a defect in early placental development, followed by generalized inflammation and progressive endothelial damage predisposing to coagulation activation. Haemostatic equilibrium shifted toward a procoagulable state in normal pregnancy is even more pronounced in P-EC, resulting in enhanced thrombin generation, increased platelet activation and deposition of microthrombi in renal and placental vasculature (4, 5). Likewise, disseminated endothelial cell dysfunction and injury occurring in P-EC have been related to the release of placenta-derived factors and their effects on the maternal vasculature. The maternal circulating concentrations of anti-angiogenic proteins, soluble fms-like tyrosine kinase-1 receptor (sFlt-1), and soluble endoglin (sEng) are elevated in P-EC, whereas pro-angiogenic factors, vascular endothelial growth factor (VEGF), and placental growth factor (PlGF) are reduced. The resulting angiogenic imbalance causes a maternal syndrome characterized by hypertension and proteinuria (6, 7). Furthermore, upon stimulation the endothelium expresses tissue factor (TF) and allows exposure of sub-endothelial structures, suffering loss of its non-thrombogenic features (8). Additionally, activated endothelial cells express vascular cell adhesion molecule 1 (VCAM-1) and support leukocyte adhesion, contributing to the pathological endothelial dysfunction seen in P-EC (9).

Recently, numerous studies have also reported altered numbers and phenotype of extracellular vesicles (EVs), found as potentiating factors of the prothrombotic state identified in P-EC (10). Circulating EVs are small membrane vesicles with a diameter of 0.1–1 μm, produced by cytoplasmic membrane blebbing and shedding upon cell activation or apoptosis. The most abundant originate from platelets, but EVs from different cell types are found in the blood circulation under normal physiological conditions, acting as transporters and messengers in cell to cell communication (11). Expression of cell-derived EVs associated with gestational complications, such as P-EC and recurrent pregnancy loss or fetal growth restriction, is considered as a pathogenic factor due to their procoagulatory and proinflammatory potential (12–15). The effect of EVs on the coagulation system is thought to be related to EV exposure of phosphatidylserine (PS) alone or in combination with TF, the key initiator of the blood coagulation *in vivo*. By exposing negatively charged phospholipids, EVs provide a catalytic surface that facilitates the assembly of tenase and prothrombinase complexes leading to thrombin generation and subsequent fibrin production. Concomitant expression of TF enhances the procoagulant activity of EVs, up to 2-fold, although the mechanism of TF activation and its state (truncated or untruncated) are still a debated issue (16–18). Moreover, EVs are potent proinflammatory inducers, which interact with both endothelial and immune cells, and may contribute to the widespread intravascular inflammation (19).

The aim of the present study was to measure levels of EVs and their various phenotypes in the maternal circulation of healthy pregnant women and women with P-EC, and to relate these levels to maternal global haemostatic plasma markers of coagulation activation. Additionally, we analyzed the changes in PS+ EVs populations in plasma samples from P-EC patients before and after delivery.

## Materials and Methods

### Patients

The study population consisted of 66 women at 25–39 weeks of gestation, including 36 women with a normal pregnancy and 30 women with P-EC. All investigated females were part of the larger study previously published by our group (20). We enrolled patients referred to a tertiary care maternity hospital (The Obstetrics and Gynaecology Clinic “Narodni Front”) with a confirmed diagnosis of P-EC between April 2014 and November 2016, as previously described. According to the revised criteria of the International Society for the Study of Hypertension in Pregnancy, published in 2014, diagnostic criteria for P-EC include the development of hypertension in a woman with previously normal blood pressure accompanied with one or more of the following new onset conditions: proteinuria, other maternal organ dysfunction and uteroplacental dysfunction (intrauterine growth restriction—IUGR). If the woman with chronic hypertension also manifests one or more of the above features of P-EC, this is classified as chronic hypertension with superimposed P-EC (21). From each patient two blood samples were collected: (1) in the morning following admission to hospital and (2) 3–10 days after delivery (mean duration 5 days).

Healthy pregnant women of similar age and gestation with no previous history of thromboembolic events, cardiovascular diseases (CVD), and/or P-EC were included as the control group. Recruitment and blood sampling were carried out during their scheduled routine prenatal care visit, with no further follow-up of pregnancy outcome.

All patients and controls gave their written informed consent and underwent an interview on their smoking habits, ongoing medication and own or family history of pregnancy complications, venous thrombotic diseases, diabetes, and CVD. The study was approved by local Ethics Committee of Gynaecology and Obstetrics Clinic “Narodni Front” in accordance with the internationally accepted ethical standards.

### Blood Sampling

Venous blood samples were collected into plastic tubes with 0.109 mol/L trisodium citrate (1 part trisodium citrate + 9 parts blood, pH 7.4). Platelet poor plasma (PPP) was obtained by double centrifugation at 2,600 g for 15 min at room temperature (with plasma supernatant harvesting in between). The final plasma supernatant was dispensed in aliquots of 500 μL and frozen at −70°C until analysis.

### Analysis of Extracellular Vesicles

The 500 μL PPP samples were thawed at 37°C for 5 min and then prepared by sequential centrifugations at 2,000 × g for 20 min and at 13,000 × g for 2 min at room temperature (with plasma supernatant harvesting in between). All measurements were performed on a Beckman Gallios flow cytometer (Beckman Coulter, Brea, CA, USA), as previously described (22). After centrifugation, 20 μl of the supernatant was incubated in the dark with 5 μl lactadherin-FITC (Haematologic Technologies, Essex Junction, VT, USA), together with either 5 μL CD42a-PE (GPIX, Beckman Coulter, Brea, CA, USA), or 5 μL CD62E-APC (E-selectin, AH diagnostics, Stockholm, Sweden), or 5 μl CD142-PE (TF, BD, NJ, USA), or 5 μL CD106-PE (VCAM-1, AH diagnostics, Stockholm, Sweden). Further phenotyping included expression of PlGF (Anti-PlGF-FITC, Abcam, Cambridge, UK). Megamix-Plus FSC (Biocytex, Marseille, France), a mix of beads with diameters (0.1, 0.3, 0.5, and 0.9 μm), was used to determine the EV gate. EVs were defined as particles <1 μm in size and positive for the antibodies described above. Lactadherin was used to identify the initial population of phosphatidylserine exposing EVs, since it is more sensitive in detection of PS-rich EVs than annexin V. The platelet and endothelial components were confirmed by their expression of CD42a and CD62E, respectively. The results are presented as concentrations of detected EVs (EVs/μl plasma).

### Global Haemostatic Assays

The EV concentrations were compared with the FVIII concentration and the results of global haemostatic assays, endogenous thrombin potential (ETP) and overall haemostatic potential (OHP), and turbidimetric parameters of fibrin clot formation, the polymerization rate (Vmax), and the number of protofibrils per fiber (Max Abs). All assays were carried out according to previously published methods (20).

### Statistical Analysis

Statistical analyses were performed using SPSS 20.0 (IBM Corp. Released 2011. IBM SPSS Statistics for Windows, Version 20.0. Armonk, NY: IBM Corp.) and R 3.4.2. (23). Depending on data distribution continuous variables are expressed as mean with standard deviation (SD) or median with inter quartile range (IQR), and compared using the parametric Student *t*-test and non-parametric Mann-Whitney *U*-test, as appropriate. Categorical variables are presented as count (%) and were compared by the Chi-square test. Pairwise comparisons were applied to compare the same index of one subject before and after delivery using the dependent samples *t*-test and the Wilcoxon Signed Rank test for variables with or without normal distribution, respectively. Correlations between independent variables were calculated using Spearman's rank correlation analysis. In order to control the analysis for confounding variables, logarithmic transformation of data was performed on several variables. Normally distributed variables were correlated using Pearson correlation analysis and partial correlation. Differences were considered significant for *p* < 0.05.

## Results

### Patient and Control Characteristics

The clinical characteristics of the study subjects are presented in Table 1. Pregnant women with P-EC and controls with uncomplicated pregnancies were of similar age, parity, and gestational age at blood sampling. There were no significant differences in smoking status or family history of CVD. However, rates of previous pregnancy complications and reported positive family history of pregnancy complications were higher in the P-EC group. Women with P-EC had a significantly higher body mass index (BMI) than control subjects (*p* < 0.001).

**Table 1:** General characteristics of patients with P-EC (*n* = 30) and controls (*n* = 36).

	**Controls**	**Patients with P-EC**	***p*-value**
Age (years)	30.6 ± 4.8	31.1 ± 6.2	0.753
BMI (kg/m^2^)	25.6 ± 2.6	30.9 ± 6.2	<0.001
Smoking status (*n*)			
- Non-smokers	30 (83%)	26 (87%)	0.745^a^
- Smokers	6 (17%)	4 (13%)	
Gestational age (weeks)	33.5 ± 3.1	33.4 ± 3.7	0.900
Parity (*n*)			
- Primiparous	18 (50%)	14 (47%)	0.810^a^
- Multiparous	18 (50%)	16 (53%)	
Previous pregnancy complications (*n*)	5 (27%)	12 (75%)	0.005^a^
Family history of pregnancy complications (*n*)			
- Positive	2 (6%)	5 (17%)	0.041^a^
- Negative	34 (94%)	25 (83%)	
Family history of CVD (*n*)			
- Positive	10 (28%)	10 (33%)	0.112^a^
- Negative	26 (72%)	20 (67%)	

*Data reported as the mean ± standard deviation or frequency n (%)*.

a *Chi-square test*.

### Extracellular Vesicles

**Figure 1:**
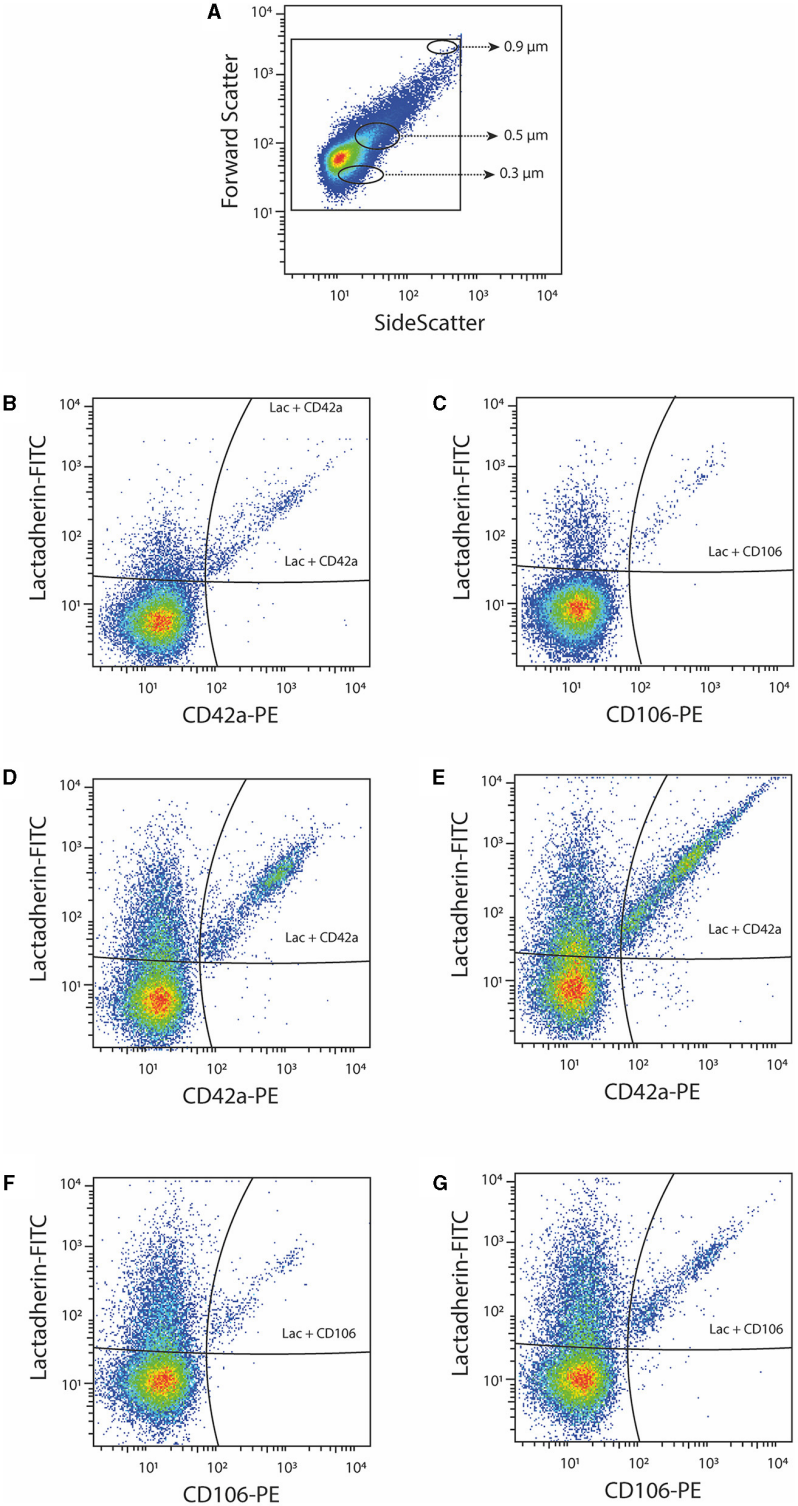
Representative dot-plots of platelet-derived (CD42a^+^) and VCAM-1^+^ (CD106^+^) EVs. **(A)** EV gating strategy based on beads with diameter 0.3, 0.5, and 0.9 μm; **(B,C)** platelet-derived (CD42a^+^) EVs and VCAM-1^+^ (CD106^+^) EVs in a healthy pregnant woman; **(D,E)** platelet-derived (CD42a^+^) EVs in a patient with pre-eclampsia before and after delivery; **(F,G)** VCAM-1^+^ (CD106^+^) EVs in a patient with pre-eclampsia before and after delivery.

PPP samples of 30 women with P-EC and 36 women with normal pregnancy were analyzed by flow cytometry and phenotyped according to protein expression. In total, 5 phenotypes of EVs were measured: PS+ CD42a^+^, PS+ CD62E^+^, PS+ CD142^+^, PS+ CD106^+^, and PlGF^+^. Figure 1 shows the gating strategy of EVs phenotyping by flow cytometry (Figure 1A) and representative dot-plots of PS+ platelet-derived EVs and PS+ VCAM-1^+^ EVs in a healthy pregnant woman (Figures 1B,C) and a patient with pre-eclampsia before and after delivery (Figures 1D–G). The largest portion of PS-exposing EVs originated from platelets in all investigated groups (36.9% in normal pregnant women, 51.5 and 29.5% in women with pre-eclampsia before and after delivery, respectively).

### Phenotyping of EVs in Women With P-EC Compared to Healthy Pregnancy

**Figure 2:**
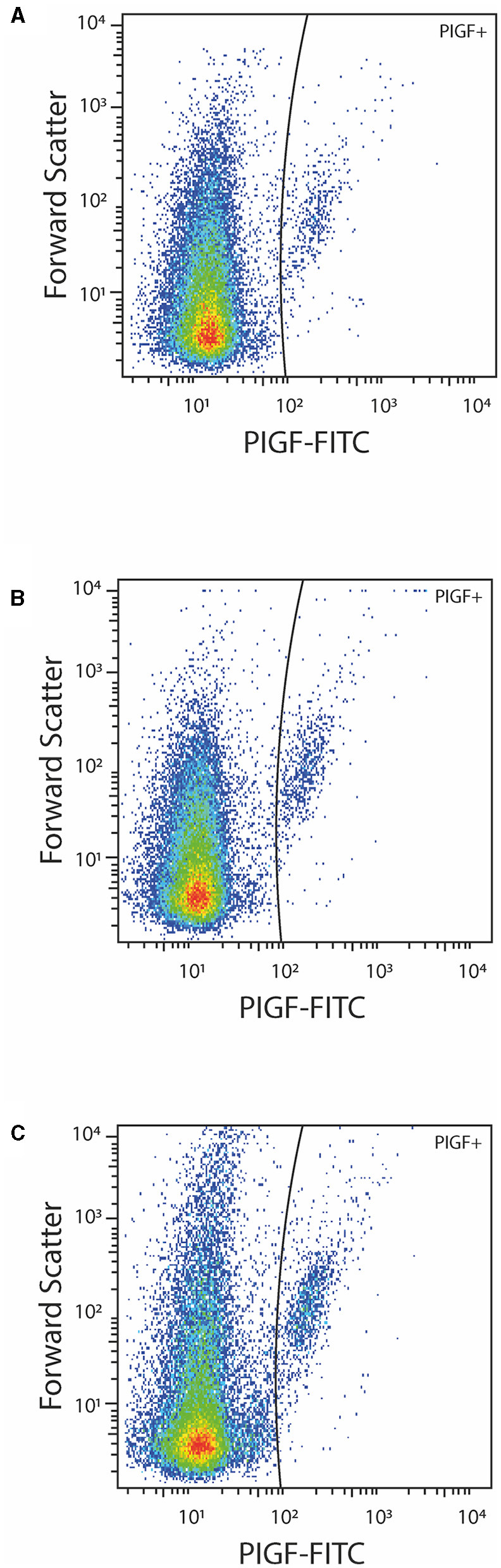
Representative dot-plots of PlGF positive EVs in a healthy pregnant women **(A)** and a patient with pre-eclampsia before **(B)** and after **(C)** delivery.

Although the concentrations of PS+ EVs in women with P-EC and healthy pregnancy were revealed to be the same, comparing different phenotypes of PS-exposing EVs we demonstrated significantly higher concentrations of PS+ CD42a^+^ platelet-derived and PS+ VCAM-1^+^ EVs in women with P-EC (Table 2). However, no differences were observed in endothelial-derived (PS+ CD62E^+^) EVs and TF-expressing PS+ EVs between P-EC patients and healthy pregnant women. Also, we found similar concentrations of PlGF-expressing EVs in the P-EC patients and healthy pregnant women (Figures 2A,B).

**Table 2:** Concentration of circulating extracellular vesicles and levels of investigated haemostatic parameters in patients with P-EC before and after delivery (*n* = 30) and in controls (*n* = 36).

**Parameter**	**Controls**	**Patients with P-EC**	
	**Before delivery**	**Before delivery**	**After delivery**
**Extracellular vesicles**			
PS+ EVs/μL	957 (586.5–2022.5)	905 (700–2100)	2445.5 (1727–4235)^†††^
PS+ CD42a^+^ EVs/μL	353 (151.5–455)	466 (327–560)^*^	720.5 (585–1266)^†††^
PS+ CD62E^+^ EVs/μL	108 (66–118)	91 (70–104)	136.5 (103–156)^†††^
PS+ CD142^+^ EVs/μL	34 (15.5–69.5)	29 (15–59)	87 (20–189)^†^
PS+ CD106^+^ EVs/μL	39.5 (27–47)	58 (40–80)^***^	102 (75–153)^†††^
PlGF^+^ EVs/μL	295.5 (176.5–408.5)	198.5 (118–327)	263 (150–399)
**Endogenous thrombin potential**			
ETP (AUC, %)	93.5 (90.5–105.0)	112.5 (106.0–119.0)^***^	107.0 (101.5–118.0)
Peak height (%)	104.8 ± 10.3	115.2 ± 13.1^**^	128.4 ± 17.9^††^
**Overall haemostatic potential**			
OCP (Abs-sum)	247.0 ± 32.9	229.4 ± 44.5	246.9 ± 50.8
OHP (Abs-sum)	177.1 ± 38.1	198.7 ± 40.7^*^	219.3 ± 43.8
OFP (%)	27.3 (16.7–35.5)	12.2 (6.6–18.8)^***^	8.8 (5.3–14.4)^†^
**Fibrin clot properties**			
Vmax (AU/min)	0.47 ± 0.14	0.55 ± 0.10^**^	0.57 ± 0.16
Max Abs (AU)	1.36 ± 0.18	1.25 ± 0.21^*^	1.30 ± 0.24
**Factor VIII activity**			
FVIII (%)	189.1 ± 78.3	259.9 ± 115.4^**^	343.2 ± 103.6^††^

*Data reported as the mean ± standard deviation or median with inter quartile range (IQR). EVs, extracellular vesicles; ETP, endogenous thrombin potential; OCP, overall coagulation potential; OHP, overall haemostatic potential; OFP, overall fibrinolysis potential; Vmax, polymerization rate; Max Abs, number of protofibrils per fiber. EV markers: CD42a, glycoprotein IX (platelet marker); CD62E, E-selectin (endothelial marker); CD142, Tissue Factor (TF); CD106, Vascular Cell Adhesion Molecule-1 (VCAM-1); PlGF, placental growth factor*.

*Controls vs. P-EC:*

* *p < 0.05,*

** *p < 0.01, and*

*** *p < 0.001.*

*P-EC before vs. after delivery:*

† *p < 0.05,*

†† *p < 0.01, and*

††† *p < 0.001*.

### Phenotyping of EVs in Women With P-EC After Delivery

The concentration of EVs and their phenotypes were also analyzed in women with P-EC before and 3–10 days after delivery. It is reasonable to expect that concentrations of EVs increase after delivery, which is associated with the delivery itself. Indeed, pairwise comparisons of results obtained in women with P-EC before and after delivery showed an ~2.5-fold increase in the concentration of PS+ EVs accompanied by the rise of all investigated EV phenotypes. As presented in Table 2, concentrations of PS+ CD42a^+^, PS+ CD62E^+^, PS+ CD142^+^, and PS+ CD106^+^ were significantly elevated in women with P-EC after delivery compared to the values before delivery. However, although slightly higher in the P-EC group after delivery, the concentration of EVs expressing PlGF did not differ significantly between P-EC patients before and after delivery (Figures 2B,C).

### Haemostatic Parameters and Their Correlation to EVs Concentrations

Results of global haemostatic assays (ETP and OHP), turbidimetric measurements of fibrin clot formation (Vmax and Max Abs), and factor VIII activity assay are presented in Table 2. Compared to gestational age-matched controls women with P-EC showed significantly elevated ETP, peak height and OHP values associated with depressed fibrinolysis [decreased overall fibrinolysis potential (OFP) values; 12.2 [6.6–18.8] % vs. 27.3 [16.7–35.5] %, *p* < 0.001]. Furthermore, P-EC patients exhibited significantly higher Vmax and lower Max Abs values, indicating the faster formation of the fibrin clot composed of thinner fibers. In the P-EC group after delivery, a significant increase of the peak height and an additionally decreased rate of fibrinolysis were observed, without significant change in fibrin clot properties. FVIII activity was above the normal range for non-pregnant individuals with a significant difference in all groups (*p* < 0.01).

Since this study focused on EVs presenting negatively charged phospholipids on their outer leaflet and their impact on coagulation disturbances, we performed Spearman correlations between the detected concentration of particles and measured haemostatic parameters (Table 3). Our results demonstrated that PS+ EVs correlated with overall coagulation potential (OCP; *r* = −0.47, *p* = 0.009), overall haemostatic potential (OHP; *r* = −0.49, *p* = 0.007) and fibrin formation parameters: maximum absorbance (Max Abs; *r* = −0.49, *p* = 0.011) and polymerization rate (Vmax; *r* = −0.41, *p* = 0.031). The same coagulation parameters correlated also with the EVs copresenting TF and PS on their outer leaflet (OCP *r* = −0.44, *p* = 0.016; OHP *r* = −0.49, *p* = 0.007; Max Abs *r* = −0.46, *p* = 0.019; Vmax *r* = −0.46, *p* = 0.013, respectively). While PlGF exposing EVs also showed significant correlation with Max Abs and Vmax (*r* = −0.40, *p* = 0.045 and *r* = −0.52, *p* = 0.004, respectively), PS+ VCAM-1^+^ EVs were correlated only with FVIII activity (*r* = 0.39, *p* = 0.034). Interestingly, PS+ platelet-derived and endothelial-derived EVs showed no significant correlation with any of the investigated coagulation parameters. However, the Pearson correlation analysis showed the similar strength of association between EVs concentrations and measured haemostatic parameters, except no association was found between concentration of EVs exposing PlGF and Max Abs. After adjustment for maternal age and BMI we observed a moderate correlation between CD62E^+^ endothelial-derived EVs and ETP (*r* = −0.42, *p* = 0.030), while the association between PS+ CD106^+^ EVs concentration and FVIII activity was no longer statistically significant. Regarding maternal complications (HELLP, renal complications, thrombocytopenia, placental abruption, and neurological disorders) and perinatal complications (IUGR and oligohydramnios) observed in our P-EC group we found no significant differences in the levels of investigated EVs between patients with and without complications. Correlation analysis revealed no association between the EVs concentrations and 1- or 5-min APGAR score.

**Table 3:** Correlation between the results of haemostatic parameters and EV concentrations.

**Variable**	**PS+ EVs/μL**		**PS+ CD42a^+^ EVs/μL**		**PS+ CD62E^+^ EVs/μL**		**PS+ CD142^+^ EVs/μL**		**PS+ CD106**^+^ **EVs/μL**		**PlGF**^+^ **EVs/μL**	
	**Rho**	***p***	**Rho**	***p***	**Rho**	***p***	**Rho**	***p***	**Rho**	***p***	**Rho**	***p***
ETP	−0.18	0.36	−0.07	0.71	−0.20	0.29	−0.32	0.09	−0.05	0.78	−0.32	0.09
Peak height	−0.01	0.99	0.22	0.24	−0.12	0.53	−0.27	0.14	0.11	0.56	−0.16	0.39
OCP	−0.47	**0.009**	−0.14	0.47	−0.24	0.20	−0.44	**0.016**	0.01	0.99	−0.35	0.058
OHP	−0.49	**0.007**	−0.17	0.39	−0.24	0.22	−0.49	**0.007**	−0.07	0.72	−0.36	0.053
OFP	−0.10	0.58	0.00	0.99	−0.12	0.51	0.23	0.22	−0.07	0.71	0.10	0.59
Vmax	−0.41	**0.031**	−0.20	0.31	−0.28	0.14	−0.46	**0.013**	−0.23	0.24	−0.52	**0.004**
Max Abs	−0.49	**0.011**	−0.28	0.16	−0.28	0.17	−0.46	**0.019**	0.07	0.74	−0.40	**0.045**
FVIII	0.12	0.54	0.18	0.33	0.21	0.27	0.03	0.88	0.39	**0.034**	0.09	0.63

*All the data were assessed using Spearman's rank correlation analysis and are reported as the correlation coefficients (Rho) with the corresponding p-values (p). ETP, endogenous thrombin potential; OHP, overall haemostatic potential; OCP, overall coagulation potential; OFP, overall fibrinolysis potential; Vmax, polymerization rate; Max Abs, number of protofibrils per fiber; FVIII, factor VIII activity; EVs, extracellular vesicles. EV markers: CD42a, glycoprotein IX (platelet marker); CD62E, E-selectin (endothelial marker); CD142, Tissue Factor (TF); CD106, Vascular Cell Adhesion Molecule-1 (VCAM-1); PlGF, placental growth factor. All significant associations are bolded*.

## Discussion

Our findings indicate that pre-eclamptic pregnancy is associated with significantly higher plasma levels of PS+ platelet-derived EVs expressing CD42a and PS+ VCAM-1^+^ EVs in comparison with normotensive pregnancy. Moreover, P-EC patients after delivery had markedly elevated concentrations of PS+ CD42a^+^ EVs, CD62E^+^ EVs, TF^+^ EVs, and VCAM-1^+^ EVs compared to those before delivery, but there was no evidence of increased PlGF^+^ EVs concentration. Haemostatic results confirmed the presence of the pronounced hypercoagulable state in P-EC patients in comparison with healthy pregnant women. Patients with P-EC exhibited enhanced thrombin generation, characterized by elevated ETP and peak height values, accompanied by elevated OHP and reduced OFP values implying reduced fibrinolytic capacity. Moreover, a higher polymerization rate (Vmax) and lower Max Abs values indicating the faster formation of condensed fibrin clots composed of thinner fibers revealed altered fibrin clot properties in this specific group of patients. Further increase in peak height combined with a decrease in OFP values was recorded in P-EC patients after delivery suggesting the presence of activated coagulation and diminished fibrinolysis despite the cessation of pregnancy. To address the role of PS+ EVs in the hypercoagulable state present in P-EC, we analyzed their association with global haemostatic assays (ETP and OHP), as well as with fibrin clot properties. A moderate inverse association was observed between EVs concentration (PS+, PS+ TF^+^, and PlGF^+^) and OHP, OCP, and fibrin formation parameters, while PS+ VCAM-1^+^ EVs directly correlated with FVIII activity in plasma. Surprisingly, PS+ EVs originating from platelets and endothelial cells did not show a correlation with any of the investigated coagulation parameters, suggesting other possible contributions to the hypercoagulable state present in P-EC.

As expected and in line with previous reports, our results revealed no difference between women with P-EC and control subjects in concentration of PS+ EVs (24, 25). However, the focus of this study was to investigate the expression of procoagulant PS on EVs, and unlike most others that used annexin V to detect PS, we employed lactadherin, which has been shown to bind PS more efficiently in a calcium-independent manner (26). In our study, both absolute and relative levels of PS+ CD42a^+^ platelet-derived EVs (GPIX, adhesive platelet marker) were significantly elevated in patients with P-EC, although showing no correlation to thrombin generation, fibrin formation and degradation, or increased fibrin network density. Studies evaluating the levels of platelet-derived EVs during pregnancy complicated by P-EC have given conflicting results, with either reduced or unchanged, and even elevated platelet-derived EVs levels between P-EC and healthy pregnant women, but none of them used the CD42a marker (14, 27, 28). Lack of direct association between PS+ CD42a^+^ platelet-derived EVs and investigated coagulation parameters in our study suggests that PS alone may not be sufficient to facilitate thrombus formation. VanWijk et al. reported that EVs induced the TF/FVII-dependent coagulation activation pathway but did not enhance thrombin generation and therefore concluded that EVs were not directly involved in the increased coagulation activation in P-EC (29). Recent *in vitro* studies implied the participation of platelet-derived EVs in coagulation propagation via TF- or contact-dependent thrombin generation, but could not demonstrate their impact on fibrin network density or stability (30, 31). Nevertheless, in the inflammation setting, as seen in P-EC, TF derived from stimulated monocytes in the circulation may act as initiator. Moreover, the interaction of platelet-derived EVs with leukocytes or endothelial cells may activate these cells and induce their TF-dependent procoagulant activity as well as further increase inflammatory reactions (32). In addition, platelet-derived EVs, by complement activation, may be further involved in the regulation of clot structure and function. P-EC is associated with abnormal complement activation while an excessive activation of the terminal pathway has been described in P-EC complicated by IUGR. Therefore, systemic activation of complement system might have an important input to the coagulation and inflammation disturbances bridging them to the maternal syndrome of P-EC (33–35).

Hypercoagulability, platelet activation, and inflammation are systemic manifestations accompanying maternal hypertension and proteinuria, as clinical hallmarks of P-EC (36). However, the primary disorder has been related to placental ischemia and oxidative stress leading to endothelial activation and injury. Endothelial cell activation could be associated with elevated levels of endothelial-derived EVs and several studies found significantly elevated concentration of endothelial-derived EVs in patients with P-EC (27, 37, 38). Our results showed no difference between the P-EC and control groups in concentrations of PS+ endothelial-derived EVs or their association with investigated coagulation parameters. As endothelial-derived EVs could express procoagulant, anticoagulant, and fibrinolytic activity, these findings might reflect their ambivalent role in coagulation and fibrinolysis (39–41). However, here we report for the first time in P-EC patients, a significantly increased concentration of PS+ EVs expressing VCAM-1. VCAM-1 has been proposed as a pro-inflammatory marker, being expressed exclusively on cytokine-stimulated endothelium and promoting firm adhesion of mononuclear cells to endothelium. Increased levels of soluble VCAM-1 in the plasma have been previously reported in patients with P-EC, vascular and inflammatory diseases, while elevated VCAM-1^+^ EVs were demonstrated only in the latter (42–46). Additionally, in our study PS+ VCAM-1^+^ EVs levels correlated significantly with FVIII activity, suggesting that PS+ EVs with increased VCAM-1 exposure are more coagulable and potentially more prone to cell interaction. Nevertheless, further studies are needed to elucidate if an increase in PS+ VCAM-1^+^ EVs could be attributed to changes in endothelium function that may potentiate a hypercoagulable state.

Upregulated TF expression on endothelial cells following endothelial stimulation or inflammatory-induced on the surface of circulating monocytes, tissue macrophages, and neutrophils has also been associated with release of TF-bearing EVs into the blood circulation and observed in different diseases (47). Concurrent expression of PS and TF on EVs potentiate the procoagulant effect of EVs. In line with previous studies, we found similar levels of PS+ TF^+^ EVs in both the P-EC and the control group, while a significant increase was observed in the P-EC group after delivery (12, 29). A post-delivery rise in TF-dependent coagulation has been described (48). However, not MP-bound TF, but soluble TF was measured in this study.

Although numerous investigations showed that the endothelial dysfunction present in maternal tissue of P-EC patients is associated with decreased plasma concentrations of PlGF, due to its binding to excessively released sFlt-1 (49), we observed no significant change in the concentration of PlGF^+^ EVs in the investigated groups (Figure 2). A clear conclusion about our findings with regard to PlGF-exposing EVs and their associations with haemostatic parameters could not be drawn since we did not explore the cellular origin of these circulating PlGF^+^ EVs, nor measure the PlGF concentration in plasma. Bearing in mind that PlGF is a more sensitive and precise predictor of P-EC than any other single biomarker, possible implications of such an observation remain to be determined.

Interestingly, concentrations of PS+, PS+ TF^+^, and PlGF^+^ EVs inversely correlated with OHP, OCP, and fibrin formation parameters assessed in the evaluation of haemostatic status in patients with P-EC. Our results are consistent with a recent report suggesting that EVs may be incorporated in the haemostatic plug and therefore inversely associated with haemostatic activation (50). Nevertheless, a significant increase of EVs concentration in P-EC patients after delivery, without further change in OHP, OCP, Vmax and Max Abs, may suggest a possible additional anticoagulant effect of high levels of PS+ TF^+^ EVs through thrombin-catalyzed activation of protein C (APC) orchestrated by soluble thrombomodulin (TM). Increased levels of TM, released due to diffuse endothelial damage, have been observed in P-EC (51, 52). However, this presumption of the potential anticoagulant effect of EVs should be studied further. In the above-mentioned study (50), EV concentration further correlated directly with Ks (coefficient of fibrin clot permeability), indicating an inverse correlation to fibrin tightness demonstrated also (via Vmax and Max Abs) in our study.

The present investigation has certain limitations. The number of patients included in the evaluation was small. Additionally, we were not able to compare data obtained in P-EC patients after delivery with post-delivery data of healthy pregnant women due to the study design.

In conclusion, numbers of CD42a platelet and VCAM-1 positive PS-exposing EVs were increased in P-EC patients before and after delivery, accompanied by additional post-partum elevation of the total, endothelial and TF-bearing PS+ EVs. Inverse association of PS+ and TF^+^ PS+ EVs with OHP and fibrin clot properties may suggest EVs involvement in intravascular fibrin deposition leading to the consequent microcirculation disorders found in P-EC. Furthermore, EVs contribution to unfavorable clot features combined with the engagement of PS+ VCAM-1^+^ EVs in P-EC may be associated with subsequent CVD development in these patients.

## Data Availability Statement

The original contributions presented in the study are included in the article, further inquiries can be directed to the corresponding author.

## Ethics Statement

The studies involving human participants were reviewed and approved by local Ethics Committee of Gynaecology and Obstetrics Clinic Narodni Front, Belgrade, Serbia. The patients/participants provided their written informed consent to participate in this study.

## Author Contributions

SL-C, VD, MK, FM, and AA were responsible for the study conception and design, interpreted the data, and critically revised and finalized the manuscript. SL-C performed global haemostatic assays, analyzed the data, and drafted the manuscript. FM designed the flow cytometric panels, analyzed the EV samples, and prepared figures. VM-M and ZM recruited participants, contributed to data collection, and interpretation of the results. All authors reviewed and approved the final version of the manuscript.

## Funding

This study was performed by grants provided by Region Stockholm (ALF project), King Gustaf the V-80 years foundation and the Swedish Rheumatism Association.

## Conflict of Interest

The authors declare that the research was conducted in the absence of any commercial or financial relationships that could be construed as a potential conflict of interest.

## Publisher's Note

All claims expressed in this article are solely those of the authors and do not necessarily represent those of their affiliated organizations, or those of the publisher, the editors and the reviewers. Any product that may be evaluated in this article, or claim that may be made by its manufacturer, is not guaranteed or endorsed by the publisher.
